# High risk of malnutrition is associated with low muscle mass in older hospitalized patients - a prospective cohort study

**DOI:** 10.1186/s12877-017-0505-5

**Published:** 2017-06-05

**Authors:** Vincent D. Pierik, Carel G. M. Meskers, Jeanine M. Van Ancum, Siger T. Numans, Sjors Verlaan, Kira Scheerman, Roeliene C. Kruizinga, Andrea B. Maier

**Affiliations:** 1 Department of Internal Medicine, Section of Gerontology and Geriatrics, VU University Medical Center, Amsterdam, The Netherlands; 2 Department of Rehabilitation Medicine, VU University Medical Center, Vrije Universiteit De Boelelaan 1105, 1081HV Amsterdam, The Netherlands; 3 Department of Human Movement Sciences, MOVE Research Institute Amsterdam, VU University, Amsterdam, The Netherlands; 4 Nutricia Research, Nutricia Advanced Medical Nutrition, Utrecht, The Netherlands; 5 Division I, VU University Medical Center, Amsterdam, The Netherlands; 6 Department of Medicine and Aged Care, Royal Melbourne Hospital, University of Melbourne, Melbourne, Australia

**Keywords:** Hospitalization, Muscle strength, Skeletal muscle, Malnutrition, Aged, Sarcopenia

## Abstract

**Background:**

Malnutrition, low muscle strength and muscle mass are highly prevalent in older hospitalized patients and associated with adverse outcomes. Malnutrition may be a risk factor for developing low muscle mass. We aimed to investigate the association between the risk of malnutrition and 1) muscle strength and muscle mass at admission and 2) the change of muscle strength and muscle mass during hospitalization in older patients.

**Methods:**

The EMPOWER study included 378 patients aged seventy years or older who were acutely or electively admitted to four different wards of an academic teaching hospital in Amsterdam. Patients were grouped into low risk of malnutrition and high risk of malnutrition based on the Short Nutritional Assessment Questionnaire (SNAQ) score and were assessed for hand grip strength and muscle mass using hand held dynamometry respectively bioelectrical impedance analysis (BIA) within 48 h after admission and at day seven, or earlier at the day of discharge. Muscle mass was expressed as skeletal muscle mass, appendicular lean mass, fat free mass and the skeletal muscle index.

**Results:**

The mean age of the patients was 79.7 years (SD 6.39), 48.9% were female. At admission, being at high risk of malnutrition was significantly associated with lower muscle mass (Odds Ratio, 95% CI, 0.90, 0.85–0.96), but not with muscle strength. Muscle strength and muscle mass did not change significantly during hospitalization in both groups.

**Conclusion:**

In older hospitalized patients, a high risk of malnutrition is associated with lower muscle mass at admission, but not with muscle strength nor with change of either muscle strength or muscle mass during hospitalization.

## Background

The physiological capacity often declines with age, making older patients vulnerable to the effects of hospitalization [1, 2]. Physical inactivity during bed rest for injury or illness is a key feature during hospitalization [3]. Inactivity leads to alterations in protein synthesis and muscle breakdown which can result in loss of muscle mass, muscle strength and physical function [4, 5]. Low muscle mass is independently associated with increased morbidity and mortality [6]. Ten percent of older patients suffer from sarcopenia (i.e. low muscle mass) at hospital admission [7]. The prevalence of sarcopenia can even increase due to illness and inactivity [8].

Malnutrition is an important risk factor for developing sarcopenia and is prevalent in 56% of patients on a geriatric ward [6, 9, 10]. Parameters of malnutrition were found to be associated with both relative and absolute muscle mass in geriatric outpatients [11]. The etiology of malnutrition in older patients is usually multifactorial and includes reduced nutritional intake and metabolic effects of illness [9]. Physiological changes also play an important role as older patients may suffer from anabolic resistance, resulting in a need for higher protein intake [4, 12]. In catabolic states, which often occur during malnutrition or acute illness, skeletal muscle is prone to muscle protein dissociation [6, 8]. Malnutrition, particularly in combination with physical inactivity, may thus accelerate the process of sarcopenia which can result in serious adverse outcomes [6, 13, 14]. The extent to which malnutrition is related to muscle mass during hospitalization is not yet clear.

This study aimed to investigate the association between the risk of malnutrition, muscle strength and muscle mass at admission and change of muscle strength and muscle mass during hospitalization in older patients dependent on the risk of malnutrition.

## Methods

### Design and patients

The Evaluation of Muscle parameters in a Prospective cohort of Older patients at clinical Wards Exploring Relations with bed rest and malnutrition (EMPOWER) study is an observational, prospective, longitudinal inception cohort study. 838 Patients aged 70 years or older who were admitted to one of four clinical wards (acute admission, internal medicine, neurosurgery and orthopedics or traumatology) of the VU University Medical Center, Amsterdam, the Netherlands in the period from April 2015 to December 2015 were considered eligible and subsequently screened for participation in EMPOWER.

**Fig. 1:**
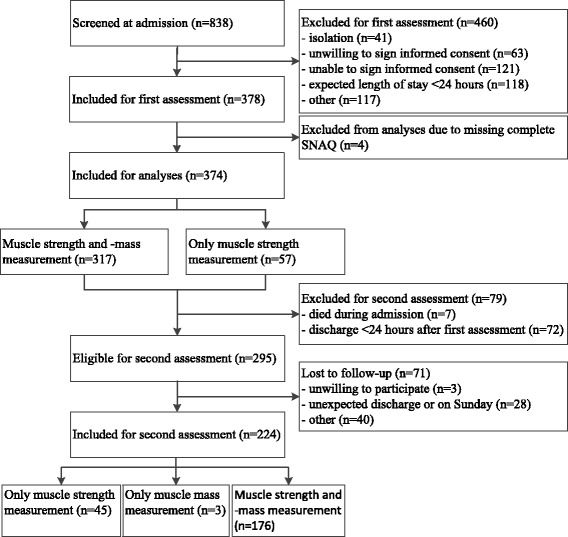
Flowchart of patients included for each assessment

Patients had to sign informed consent to participate in this study. Patients were excluded if: (i) their expected length of stay was less than 24 h; (ii) they were nursed in isolation rooms; (iii) they were terminally ill; (iv) they were not able to understand the Dutch language. Finally, 378 patients were included in the EMPOWER study (see Fig. 1). Patients were assessed at two occasions during their admission, i.e. within 48 h after admission, at the day of discharge or at day 7 after the first assessment if patients were still in hospital. If patients were discharged within 24 h after the first assessment, they were excluded from follow-up. 224 Patients (59%) were assessed at two occasions during their admission. The study design was approved by the research ethics committee of the VU University Medical Center, Amsterdam, The Netherlands.

### Determinants and outcome measures

Data collected from medical records included socio-demographics, number of medications, number of comorbidities and attending medical specialism. Data that were gathered during an interview with the patient included alcohol use, smoking habits, use of a walking aid, living situation and falls during the last six months. Weight was measured on a weighing chair.

If patients were unable to get out of bed, an estimate was obtained from the patient or relative. Height was estimated using knee-height and the Longitudinal Aging Study Amsterdam formula (LASA): female: height (cm) = 68.74 – (0.16 x age) + (2.07 x knee-height in cm), male: height (cm) = 74.48 – (0.15 x age) + (2.03 x knee-height in cm) [15]. Functional performance was assessed by the Katz index of independence in Activities of Daily Living (range 0–6) [16]. Cognition was scored with the 6-item cognitive impairment test (range 0–28), a brief and simple test of cognition [17]. A numeric pain rating scale was used to assess pain in the patients (range 0–10) [18]. Functional ambulation classification was used to classify mobility (range 0–5) [19]. Brown’s in-hospital mobility rating was used to rate physical activity during hospitalization (range 0–12) [20]. According to this rating; patients who walked at least two times a day were considered as a high-mobility group (median score > 8).

#### Risk of malnutrition

Risk of malnutrition was determined using the Short Nutritional Assessment Questionnaire (SNAQ) score (range 0–7). The SNAQ is an easy, valid and reproducible questionnaire for early detection of hospital malnutrition including questions about unintentional weight loss, decrease in appetite over the last month and the use of supplemental drinks or tube feeding over the last month [21]. Four out of 378 patients were excluded from further analyses because of an incomplete SNAQ score. Patients were grouped into low risk of malnutrition (SNAQ-score 0–1) and high risk of malnutrition (SNAQ-score ≥ 2). Screening for malnutrition by use of the SNAQ-score was part of regular care. Following the standard hospital care, patients at moderate risk of malnutrition (i.e. SNAQ-score 2) are offered energy- and protein rich meals and patients at severe risk of malnutrition (i.e. SNAQ-score > 2) are offered a dietary intervention. Including energy- and protein rich meals, supplementation or tube feeding.

#### Muscle strength

Muscle strength was measured by a Jamar dynamometer for hand grip strength (HGS) in a sitting position with elbows flexed at 90 degrees, shoulders adducted and forearms in neutral position without support. If patients were unable to get out of bed, HGS was measured with the bed in an angle of approximately 30 degrees and the elbows unsupported. Patients were actively encouraged to squeeze maximally. Both hands were assessed and two attempts were allowed per hand. The maximum score of either the left or the right hand was noted. At the second assessment patients were asked to take the same position as during the first assessment.

#### Muscle mass parameters

Muscle mass parameters were measured using a multi frequency bioelectrical impedance analyzer (BIA) (InBody S10, Biospace). Multi frequency BIA analysis is a valid tool for the assessment of body-composition and segmental lean measurements [22] and a good portable alternative to dual energy X-ray absorptiometry (DEXA), which is often used as a reference method [23]. Patients were asked to lie down in supine position with straightened arms and legs whenever able and to lie as still as possible during the measurement. Patients who were unable to lie down were measured in a sitting position with straightened arms and the BIA analyzer set to seated posture. Skeletal muscle mass, appendicular lean mass and fat free mass were noted. A distinction was made between absolute and relative values. Relative values of muscle-, appendicular lean- and fat free mass were calculated by dividing the value by total body weight and multiplying it with 100%. The skeletal muscle index was calculated by dividing skeletal muscle mass by squared height in meters [24]. In case of an implantable cardioverter defibrillator or other implanted devices (*n* = 29) or if it was impossible to position the electrodes at both middle fingers, thumbs and ankles (*n* = 28) BIA was not assessed.

### Statistical analyses

To find a statistically significant difference of 0.5 kg/m^2^ decrease in skeletal muscle index between two measurements with a power of 80% and an estimated standard deviation of 2.5 [25], 197 subjects had to be included.

Statistical Package for the Social Sciences (IBM SPSS Statistics for Windows, Version 23.0. Armonk, NY, IBM Corp) was used for analyses. Data with a skewed distribution were presented as median and interquartile range.

A logistic regression analysis was performed to analyze associations between the risk of malnutrition, muscle strength and muscle mass parameters (i.e. muscle mass, appendicular lean mass and fat free mass) at admission. Analyses were adjusted for age and sex (model 1) and additionally for comorbidities (model 2). To account for difference in body composition, absolute muscle mass parameters (skeletal muscle mass, appendicular lean mass and fat free mass) were additionally adjusted for relative muscle mass parameters at admission and relative muscle mass parameters (relative skeletal muscle mass, relative appendicular lean mass and relative fat free mass) for weight at admission (model 3).

Paired samples t-tests were used to analyze changes of muscle strength and muscle mass parameters during hospitalization on significance in both groups. A logistic regression analysis was performed to analyze the associations between the risk of malnutrition, the change of muscle strength and muscle mass parameters during hospitalization. These analyses were adjusted for age, sex, time between the measurements and the corresponding value at admission (model 1). The other adjustment models were identical to the cross-sectional analyses. *P*-values below 0.05 were considered statistically significant.

## Results

**Fig. 2:**
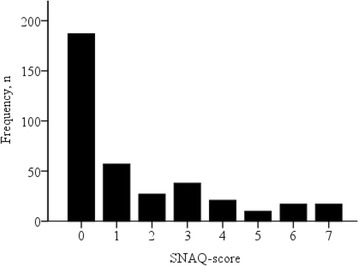
Histogram of the Short Nutritional Assessment Questionnaire-Score of the Patients

Table 1 shows the characteristics of the study population. The mean age was 79.7 years (SD 6.39) and 48.9% of the patients were female. At admission, 34.8% of the patients were at high risk of malnutrition according to the SNAQ (Fig. 2).

**Table 1:** Patient characteristics of the entire cohort and stratified by the risk of malnutrition

			Risk of malnutrition	
	*N*	All	Low	High
			*n* = 244	*n* = 130
Age, years, mean (sd)	374	79.7 (6.39)	79.3 (6.19)	80.3 (6.74)
Sex, female	374	183 (48.9)	123 (50.4)	60 (46.2)
Living independently	373	327 (87.7)	217 (88.9)	110 (85.3)
Weight, kg, mean (sd)	374	73.2 (17.1)	75.4 (18.0)	69.1 (14.4)
Height, cm, mean (sd)	374	169 (9.46)	168 (9.17)	169 (9.99)
BMI, kg/m^2^, mean (sd)	374	25.8 (5.77)	26.6 (5.92)	24.2 (5.15)
Current smoking	365	39 (10.7)	29 (12.2)	10 (7.8)
Alcohol use	364	146 (40.1)	112 (47.5)	34 (26.6)
Elective admission	374	58 (15.5)	47 (19.3)	11 (8.5)
Admission, non-surgical	374	205 (54.8)	115 (47.1)	90 (69.2)
Brown’s-score > 8	331	138 (41,7)	90 (41,7)	48 (41,7)
LOS, days, median (IQR)	374	5.0 (2.9–7.8)	4.9 (2.8–7.4)	5.2 (3.0–9.4)
Number of medications >4	374	230 (61.5)	140 (57.4)	90 (69.2)
Number of comorbidities >1	372	329 (88.4)	214 (88.1)	115 (88.5)
KATZ ADL-score > 1	370	149 (40.3)	93 (38.4)	56 (43.8)
6-item CIT, median (IQR)	366	4 (0–8)	4 (0–8)	4 (0–10)
NRS-score on pain, median (IQR)	370	2 (0–5)	2 (0–6)	1 (0–5)
FAC-score > 0	370	273 (73.8)	166 (68.9)	107 (82.9)
Use of walking aid	372	198 (53.2)	125 (51.4)	73 (56.6)
Fallen last six months	374	169 (45.2)	118 (48.4)	51 (39.2)
Time between measurements, days, median (IQR)	224	5.0 (3.0–7.0)	4.9 (3.0–6.9)	6.0 (3.9–7.0)

All variables are presented as *n* (%) unless indicated otherwise. All variables were measured at baseline, except for length of stay and time between measurements

Brown’s in-hospital mobility rating (range 0–12). LOS Length Of Stay. KATZ-ADL Katz Index of Independence in Activities of Daily Living (range 0–6). 6-item CIT 6-item Cognitive Impairment Test (range 0–28). NRS Numerical Rating Scale (range 0–10). FAC Functional Ambulation Classification (range 0–5)

Table 2 shows the association of muscle strength and muscle mass parameters dependent on the risk of malnutrition at admission. The risk of malnutrition was not associated with muscle strength. High risk of malnutrition was significantly associated with lower absolute skeletal muscle-, appendicular lean- and fat free mass (Odds Ratios respectively 0.90, 0.89 and 0.95). None of the relative parameters of skeletal muscle-, appendicular lean- or fat free mass was associated with the risk of malnutrition.

**Table 2:** Hand grip strength and muscle mass parameters dependent on the risk of malnutrition

			Risk of malnutrition		Model 1		Model 2		Model 3	
			Low	High	OR	95% CI	OR	95% CI	OR	95% CI
			*n* = 244	*n* = 130						
HGS, kg	♂	191	27.1 (10.3)	24.3 (8.91)	0.98	0.95–1.01	0.98	0.95–1.01	NA	
	♀	183	15.1 (5.75)	14.8 (5.35)						
SMM, kg		317	26.8 (6.12)	25.1 (5.55)	0.92	0.87–0.97	0.92	0.86–0.97	0.90	0.85–0.96
ALM, kg		317	20.6 (5.29)	19.4 (5.78)	0.94	0.89–0.99	0.94	0.88–0.99	0.89	0.83–0.96
FFM, kg		317	49.9 (10.5)	47.2 (9.61)	0.96	0.93–0.99	0.95	0.92–0.99	0.95	0.92–0.98
SMI, kg/m^2^	♂	157	10.1 (1.39)	9.13 (1.43)	0.69	0.57–0.83	0.68	0.56–0.83	NA	
	♀	160	8.79 (1.24)	8.41 (1.24)						
Relative SMM, %		317	36.1 (5.81)	37.0 (6.25)	1.03	0.99–1.07	1.03	0.98–1.07	0.98	0.93–1.04
Relative ALM, %		317	27.6 (4.74)	28.6 (7.37)	1.03	0.99–1.09	1.03	0.99–1.08	1.01	0.96–1.06
Relative FFM, %		317	67.5 (10.4)	69.9 (11.5)	1.02	1.00–1.05	1.02	1.00–1.05	1.00	0.97–1.03

All variables are presented as mean (sd)

HGS Hand Grip Strength. SMM Skeletal Muscle Mass. ALM Appendicular Lean Mass. FFM Fat Free Mass. SMI Skeletal Muscle Index. NA Not Applicable. Model 1 adjusted for age and sex. Model 2 as 1 and comorbidities. Model 3 as 1 and absolute muscle parameters (SMM, ALM, FFM) for corresponding relative muscle parameter at admission, relative muscle parameters for weight at admission

Table 3 shows the changes of muscle strength and muscle mass parameters during hospitalization stratified by the risk of malnutrition. No significant changes of muscle strength, absolute muscle- or fat free mass were found. In both, the low-risk group and the high-risk group, absolute and relative appendicular lean mass increased significantly during hospital stay (respectively 0.8 kg and 1.4%). Controlling the analyzes for volemic status based on clinical symptoms i.e. edema and skin turgor did not affect the results (data not shown). Relative fat free mass did increase significantly in the high-risk group (1.6%). No differences in the change of muscle strength and muscle mass parameters during hospitalization were found dependent on the risk of malnutrition (Table 4).

**Table 3:** Change of hand grip strength and muscle mass parameters stratified by the risk of malnutrition

			Risk of malnutrition					
			Low *n* = 159			High *n* = 65		
			Admission	In-hospital follow up	P	Admission	In-hospital follow up	P
HGS, kg	♂	101	26.4 (9.64)	27.5 (9.63)	0.082	24.0 (9.34)	24.8 (8.69)	0.223
	♀	120	15.2 (5.44)	15.9 (5.24)	0.069	13.0 (4.65)	14.2 (4.94)	0.115
SMM, kg		179	25.7 (5.36)	25.6 (5.47)	0.455	24.5 (5.75)	24.8 (5.60)	0.270
ALM, kg		179	19.6 (4.77)	20.0 (4.87)	0.030	18.7 (5.19)	19.5 (5.30)	0.007
FFM, kg		179	48.1 (9.20)	48.2 (9.43)	0.678	46.2 (10.0)	47.1 (9.96)	0.088
SMI, kg/m^2^	♂	78	9.90 (1.21)	9.83 (1.23)	0.404	9.00 (1.65)	9.09 (1.65)	0.470
	♀	101	8.67 (1.18)	8.62 (1.33)	0.683	8.46 (1.27)	8.58 (1.07)	0.411
Relative SMM, %		179	36.6 (5.59)	36.3 (5.62)	0.380	36.8 (6.73)	37.4 (7.24)	0.128
Relative ALM, %		179	27.6 (4.37)	28.2 (4.82)	0.027	27.9 (5.54)	29.3 (6.81)	0.002
Relative FFM, %		179	68.5 (10.1)	68.6 (10.3)	0.800	69.7 (12.6)	71.3 (13.8)	0.038

All variables are presented as mean (sd). HGS Hand Grip Strength. SMM Skeletal Muscle Mass. ALM Appendicular Lean Mass. FFM Fat Free Mass. SMI Skeletal Muscle Index

**Table 4:** Differences in change of hand grip strength and muscle mass parameters dependent on the risk of malnutrition

			Risk of malnutrition		Model 1		Model 2		Model 3	
			Low	High	OR	95% CI	OR	95% CI	OR	95% CI
			*n* = 159	*n* = 65						
HGS, kg	♂	101	+1.05 (4.77)	+0.75 (3.62)	1.01	0.93–1.09	1.01	0.93–1.09	NA	
	♀	120	+0.78 (4.00)	+1.23 (4.20)						
SMM, kg		179	−0.14 (2.09)	+0.28 (1.86)	1.05	0.89–1.23	1.05	0.89–1.23	1.05	0.89–1.24
ALM, kg		179	+0.38 (1.93)	+0.80 (2.08)	1.05	0.88–1.24	1.05	0.88–1.24	1.05	0.88–1.24
FFM, kg		179	+0.14 (3.81)	+0.85 (3.55)	1.02	0.93–1.12	1.02	0.93–1.12	1.03	0.94–1.12
SMI, kg/m^2^	♂	78	−0.06 (0.56)	+0.09 (0.61)	1.06	0.68–1.66	1.06	0.68–1.66	NA	
	♀	101	−0.04 (0.92)	+0.12 (0.76)						
Relative SMM, %		179	−0.25 (3.16)	+0.60 (2.84)	1.07	0.96–1.19	1.07	0.96–1.19	1.06	0.95–1.18
Relative ALM, %		179	+0.58 (2.88)	+1.44 (3.24)	1.06	0.95–1.19	1.06	0.95–1.19	1.06	0.94–1.18
Relative FFM, %		179	+0.13 (5.73)	+1.58 (5.39)	1.03	0.98–1.10	1.03	0.98–1.10	1.03	0.97–1.09

All variables are presented as mean (sd). HGS Hand Grip Strength. SMM Skeletal Muscle Mass. ALM Appendicular Lean Mass. FFM Fat Free Mass. SMI Skeletal Muscle Index. NA Not Applicable. Model 1 adjusted for age, sex, time between the two measurements and value at admission. Model 2 as 1 and comorbidities. Model 3 as 1 and absolute muscle parameters (SMM, ALM, FFM) for corresponding relative muscle parameter at admission, relative muscle parameters for weight at admission

## Discussion

In this large inception cohort of older patients during hospitalization, a high risk of malnutrition was associated with lower muscle mass but not with lower muscle strength at admission. A change of muscle strength and muscle mass during hospitalization was not associated with the risk of malnutrition.

The association of a high risk of malnutrition and lower muscle mass at admission is in line with a cross-sectional cohort study of 608 hospitalized patients with a significantly younger age compared to our cohort (median age 57 years) [26]. The prevalence of sarcopenia (i.e. low muscle mass) in that study was higher in patients that were grouped as moderately or severely malnourished based on the patient-generated subjective global assessment (PG-SGA).

To the best of our knowledge, no study has previously addressed the association between the risk of malnutrition at admission and change of muscle strength or muscle mass during hospitalization. We did expect to find a decrease of muscle mass during hospitalization due to the high prevalence of inactivity and malnutrition in older patient populations. A balance between anabolic and catabolic processes is required to maintain skeletal muscle mass [13]. Evidence shows that malnutrition can lead to a negative skeletal muscle protein balance, following muscle loss [4]. Theoretically, a week of physical inactivity increases skeletal muscle catabolism and decreases anabolism [27]. Notwithstanding, we did not find a significant decrease of skeletal muscle-, fat free mass and skeletal muscle index in the low-risk or the high-risk group. This was in line with a previous study in which no statistically significant change of fat free mass (measured by BIA) during hospitalization was found in 23 COPD patients with a mean age of 63 years [28]. In another study, a significant decrease of lean body mass was found after seven days of hospital stay in a group of 20 patients who had a median age of 70 years and underwent colorectal surgery [29]. This result may be due to low appetite, vomiting and disturbed gastrointestinal function after abdominal surgery in this selected patient population. Our study design minimized the risk of selection bias and the variety in specialisms ensured heterogeneity and a good representation of daily clinical practice.

Next to physical activity, nutrition is one of the main anabolic stimuli for muscle protein synthesis [30]. Muscle protein synthesis is driven by post-prandial plasma essential amino acid availability [27]. The patients who were at high risk of malnutrition and had a high protein intake as a result of the standard hospital care, may have had a higher muscle protein synthesis. A randomized-controlled study in 592 acutely ill older patients, not selecting on nutritional risk groups, showed that there were no significant differences in change of mid-arm circumference, triceps skinfold thickness and hand grip strength between the intervention group with additional nutritional care and the standard care group [31]. Another study in 23 hospitalized malnourished elderly patients showed a positive effect on fat free mass assessed by DXA, but not on hand grip strength, after ten days of dietary supplementation [32].

BIA measurements are relatively easy to perform with minimal burden and therefore well suited to measure body composition in vulnerable older patients. However, use of BIA may have some drawbacks as it could have been influenced by the hydration status of older patients. BIA estimates body composition by the difference in impedance of various tissues. Adipose tissue contains a relatively low amount of water compared to muscle tissue and therefore has higher impedance. A previous study of 200 acutely admitted older patients found a high prevalence of dehydration at hospital admission, which decreased during hospitalization [33]. In the same study the prevalence of malnutrition, based on the nutritional risk screening (NRS 2002), did not differ between the euhydrated and the dehydrated group. Taken together, fat tissue may have been overestimated during the measurements at admission, resulting in lower appendicular lean- and fat free mass in both the low-risk and the high-risk group. The increase of appendicular lean mass could be explained by this phenomenon. A decrease of fat free mass may have been masked in both groups.

This study included 378 participants at admission and ended up with 224 participants, of whom 179 participants with two measurements of muscle mass parameters. This implies that the study was slightly underpowered. Nevertheless, this study is the biggest until now reporting follow up data [34] and the standard deviation of skeletal muscle index was much smaller compared to the previous study [25], giving this study enough power to draw conclusions.

### Strengths and limitations

The large inception cohort of a relevant group of patients who were acute or elective admitted to different wards of surgical and non-surgical specialisms ensured heterogeneity in this study. However, the observational design did not allow us to draw conclusions concerning possible interventions. Furthermore, the use of BIA instead of magnetic resonance imaging (MRI) or computed tomography (CT) scan and the standard hospital care for patients at risk of malnutrition may have influenced our results.

## Conclusion

In older hospitalized patients, a high risk of malnutrition was significantly associated with lower absolute skeletal muscle-, appendicular lean-, fat free mass and the skeletal muscle index, but not with lower muscle strength. The risk of malnutrition was not associated with a change of these parameters during hospitalization. Further research is needed to determine the long-term impact of hospitalization on muscle mass in older patients.

## Abbreviations

6-item CIT: 6-item Cognitive impairment test
ALM: Appendicular lean mass
BIA: Bioelectrical impedance analysis
DEXA: Dual energy x-ray absorptiometry
EMPOWER: The evaluation of muscle parameters in a prospective cohort of older patients at clinical Wards Exploring Relations with bed rest and malnutrition
FAC: Functional ambulation classification
FFM: Fat free mass
HGS: Hand grip strength
KATZ-ADL: Katz index of independence in activities of daily living
NRS: Numerical rating scale
SMI: Skeletal muscle index
SMM: Skeletal muscle mass
SNAQ: Short Nutritional Assessment Questionnaire
